# Elevated plasma trimethylamine-*N*-oxide levels are associated with diabetic retinopathy

**DOI:** 10.1007/s00592-020-01610-9

**Published:** 2020-10-16

**Authors:** Weiming Liu, Chunmin Wang, Yu Xia, Wei Xia, Gaoqin Liu, Chi Ren, Yu Gu, Xin Li, Peirong Lu

**Affiliations:** 1 Department of Ophthalmology, The First Affiliated Hospital of Soochow University, 188 Shizi Street, Suzhou, 215006 People’s Republic of China; 2 Suzhou Center for Disease Control and Prevention, 72 Sanxiang Road, Suzhou, 215004 People’s Republic of China; 3 Jiangsu Key Laboratory of Clinical Immunology, Soochow University, 708 Renmin Road, Suzhou, 215006 People’s Republic of China

**Keywords:** Trimethylamine-*N*-oxide, TMAO, Gut microbiome, Metabolite, Type 2 diabetes mellitus, Diabetic retinopathy

## Abstract

**Aims:**

To determine the relationship between plasma levels of trimethylamine-*N*-oxide (TMAO) and odds of diabetic retinopathy (DR).

**Methods:**

A cross-sectional study was conducted. Blood samples were obtained from 122 type 2 diabetes mellitus (T2DM) patients with or without DR. Multivariable logistic regression analyses were performed to identify the association between plasma TMAO and DR. The diagnostic value of plasma TMAO was assessed by the area under the receiver operating characteristic curve (AUROC) and integrated discrimination improvement (IDI).

**Results:**

In the T2DM patients, plasma levels of TMAO were significantly higher in patients with DR compared with those without DR (*P* = 0.001). As logarithmic (ln) transformation of TMAO increased per standard deviation (SD), there was higher probability to have DR [odds ratio (OR) = 2.31; *P* = 0.005]. As ln-transformed TMAO increased per SD, the severity of DR was more likely to get worse (OR = 2.05; *P* = 0.004). In the diagnostic model, the addition of TMAO contributed to the improvement in AUROC from 0.646 to 0.734 (*P* = 0.043), and the IDI was 10.7% (*P* < 0.001).

**Conclusion:**

Elevated levels of plasma TMAO were associated with higher odds and worse severity of DR in T2DM patients, and further investigation is required for the causality of this association.

## Introduction

Diabetic retinopathy (DR), the ocular manifestation of end-organ damage in diabetes, is a major cause of vision impairment in middle-aged and elderly individuals [1]. The global prevalence of DR was about 34.6% among individuals with diabetes [2]. Various risk factors such as hypertension, diabetes duration and poor glycemic control have been implicated in DR [1], though few biomarkers are widely applied in clinical practice. Identification of accurate biomarkers for DR is conducive to formulate the strategies for early prevention and personalized management in the high-risk group [3].

Trimethylamine-*N*-oxide (TMAO), a gut microbiome-derived metabolite, is mainly produced from dietary L-carnitine, choline and its precursor trimethylamine (TMA). Gut microbiota transform L-carnitine and choline into TMA, which is further oxidized in liver into TMAO by flavin-dependent monooxygenase 3 (FMO3) [4]. A wealth of literature has identified plasma TMAO as a novel prognostic predictor of cardiovascular diseases (CVD) [5], and recent studies have found that non-lethal inhibition of gut microbial enzyme [6, 7] or genetic deficiency of FMO3 [8] can markedly reduce TMAO level and attenuate TMAO-induced atherosclerosis and thrombosis.

A recent meta-analysis revealed positive dose-dependent association of plasma TMAO with risk of diabetes [9]. Higher TMAO concentrations could impair glucose homeostasis and contribute to worse clinical outcome of diabetic complications [10, 11]. What’s more, FMO3 knockdown in insulin-resistant mice could prevent hyperlipidemia and hyperglycemia via decreasing plasma TMAO [12].

The evidence above implies that TMAO might be a promising biomarker for diabetes-related diseases. However, to our best knowledge, there is no reported data identifying the association between TMAO and DR. Hence, we performed this study to investigate whether plasma TMAO levels are associated with presence of DR in patients with type 2 diabetes mellitus (T2DM).

## Materials and methods

### Study population and assessment of DR

 One hundred and sixty-two inpatients at the First Affiliated Hospital of Soochow University from July 2019 to December 2019 were enrolled in this cross-sectional study. Patients of diabetes without DR (DWR) and nonproliferative DR (NPDR) were enrolled from the Department of Endocrinology, who were in hospital due to poor glycemic control and further checked fundus condition in the Department of Ophthalmology. Proliferative DR (PDR) patients and non-diabetic patients with idiopathic macular hole were enrolled from the Department of Ophthalmology, who were in hospital for vitrectomy. Participants of Chinese Han ethnicity aged 40–85 years old were defined as eligible. T2DM was diagnosed based on either the fasting plasma glucose (FPG) of ≥ 7.0 mmol/L, or glycated hemoglobin (HbA1c) ≥ 6.5% (48 mmol/mol), or a previous diagnosis of T2DM according to Standards of Medical Care in Diabetes [13]. We did not measure HbA1c of non-diabetic patients who met all of the following three criteria: FPG < 7.0 mmol/L, no symptoms of diabetes and no diabetes history. The color fundus photographs were taken with a non-mydriatic fundus camera (CR-2, Canon, Tokyo, Japan) with 45-degree field involving disk and macula. T2DM patients were further graded by two ophthalmologists into three subgroups, DWR, NPDR and PDR, regarding the condition of the worse eye. According to the international clinical diabetic retinopathy severity scales [14], NPDR was defined as the presence of microaneurysms, intraretinal hemorrhages, venous beading or intraretinal microvascular abnormalities, and PDR was defined as the presence of neovascularization or vitreous/preretinal hemorrhage. Exclusion criteria included any other ocular diseases, pregnancy and lactation, cognitive impairments, autoimmune diseases, systemic infection, terminal illness, malignancy, vegetarians and medication history of systematic antibiotics or probiotics within 3 months. The study was approved by the Ethical Committee of the First Affiliated Hospital of Soochow University and conformed to the Declaration of Helsinki. All subjects provided written informed consent prior to study participation. The registered number of this study was ChiCTR1900024138 at the Chinese Clinical Trial Registry (https://www.chictr.org.cn).

### Clinical data and biochemical analyses

Clinical data were obtained from medical records, including age, gender, duration of diabetes mellitus, history of hypertension, coronary heart disease (CHD) and diabetic nephropathy (DN). Body mass index (BMI) was calculated using weight and height measurements. Overnight fasting venous blood samples were collected on the second day morning of admission and sent to the clinical laboratory center of our hospital within 1 h for further analyses of FPG, HbA1c, serum creatinine (SCr) and lipid profiles including triglyceride, total cholesterol (TC), high density lipoprotein cholesterol (HDL-C), low density lipoprotein cholesterol (LDL-C). Estimated glomerular filtration rate (eGFR) was calculated based on Chronic Kidney Disease Epidemiology Collaboration (CKD-EPI) equation [15].

### Mass spectrometry quantification of plasma TMAO concentrations

The quantitative detection of plasma TMAO concentration was performed in Physical and Chemical Laboratory of Suzhou Center for Disease Control and Prevention by stable isotope dilution liquid chromatography–tandem mass spectrometry (LC–MS/MS) method as described elsewhere [16]. Plasma was immediately separated from fasting blood sample from the antecubital vein at 6:00 a.m. by centrifugation at 5000 rpm at 4 °C for 10 min. According to previous study [16], TMAO was stable in plasma during storage at − 80 °C for 5 years, so all plasma samples were stored at − 80 °C till being processed.

Briefly, 50 µL plasma sample was mixed with 200 µL pre-prepared internal standard, which contains 500 µg/L d_9_-TMAO (Cat. No. 791628; Sigma-Aldrich, Shanghai, China) in methanol, then followed by vortex for 30 s and centrifugation at 12,000 rpm at 4 °C for 10 min. The precipitation was discarded and the supernatant was recovered and centrifuged again in the same way. Afterward, 2 µL of supernatant was injected into ACQUITY UPLC BEH HILIC column (1.7 µm, 2.1 mm × 100 mm; Waters, Milford, MA, USA) at a flow rate of 0.2 ml/min and subsequently analyzed using Waters ACQUITY UPLC I-Class/Xevo TQD System (Waters, Milford, MA, USA). In order to elute the analytes, a gradient elution was generated by mixing solvent A (10 mmol/L ammonium acetate in water) and solvent B (acetonitrile) at following different ratios. The proportion of solvent A increased linearly from 20 to 80% within 1.8 min, maintained constant at 80% for 1.8 min, and then decreased linearly back to 20%. Electrospray ionization (ESI) in the positive ion mode was conducted with multiple reaction monitoring (MRM) using characteristic precursor–product ion transitions as follows: TMAO at m/z 76 → 58 and d_9_-TMAO at m/z 85 → 66. A standard curve was established by using a serial dilution of TMAO standard (Cat. No. 317594; Sigma-Aldrich, Shanghai, China) mixed with a fixed concentration of d_9_-TMAO (Cat. No. 791628; Sigma-Aldrich, Shanghai, China) and the correlation coefficient (*r*^2^) was 0.999. The peak area ratio of TMAO and d_9_-TMAO was used to quantify TMAO concentration with MassLynx v4.1 software (Waters, Milford, MA, USA). All of the assays were performed blind.

### Statistical analyses

All statistics were performed using the SPSS 24.0 software (IBM Corp., Armonk, NY, USA) and statistical significance was set at *P* < 0.05 (two-tailed). Continuous variables were expressed as mean with standard deviation (SD) or median with interquartile range (IQR) based on normality of data distribution assessed by Shapiro–Wilks test. We applied a logarithmic (ln) transformation of TMAO values to obtain normally distributed data [17]. Comparison between two groups was performed using Mann–Whitney* U* test for nonparametrically distributed data. Differences among multiple groups were analyzed using one-way ANOVA and Kruskal–Wallis test for means and medians, respectively, and Bonferroni post hoc pairwise comparison was performed when necessary. For categorical variables, data were presented as number with percentage and compared using Chi-Square analysis. Spearman rank correlations were analyzed between levels of plasma TMAO and other continuous variables in T2DM patients. Tukey box and whisker plots were graphed using GraphPad Prism Version 8.0 (GraphPad Software, Inc., San Diego, CA, USA).

To identify the association between plasma levels of TMAO and DR in T2DM patients, we used binary logistic regression to estimate the odds ratios (ORs) and 95% confidence intervals (CIs) for DR across quartiles of plasma TMAO and per SD increment in the ln-transformed TMAO [17]. We further identified this association in different subgroups and inferred a subgroup effect through evaluation of the *P* value for interaction. Furthermore, ordinal logistic regression was employed to determine the relationship between plasma TMAO concentrations and DR severity. In multivariable logistic regression models, we adjusted age and gender in Model 1, and further adjusted BMI, diabetes duration and hypertension in Model 2. To assess whether this association was influenced by renal function, we further adjusted eGFR in Model 3.

The area under the receiver operating characteristic curve (AUROC) was generated by MedCalc Version 19.0.7 (MedCalc, Mariakerke, Belgium) to assess the value of TMAO in diagnosing DR. The optimal cutoff value was determined using the maximal *Youden* index. The primary diagnostic model involved the widely known risk factors for DR, including age, gender, BMI, diabetes duration and hypertension, and we then compared the AUROC before and after adding TMAO as a new factor into the model. Besides, integrated discrimination improvement (IDI) was estimated as well to assess the additional effect of TMAO in diagnosing DR [18].

## Results

### Characteristics of the study participants

The study population consisted of 122 T2DM patients (50 without DR, 30 with NPDR and 42 with PDR) and 40 non-diabetic patients (set as control). As shown in Table 1, there are no significant differences in age, gender, BMI, diabetes duration, history of hypertension, CHD, DN, SCr, eGFR and lipid profiles across different groups. Curiously, PDR patients had lower concentrations of FPG and HbA1c than those in DWR and NPDR groups.

**Table 1:** Basic and metabolic characteristics of the study participants

	Control (*N* = 40)	DWR (*N* = 50)	NPDR (*N* = 30)	PDR (*N* = 42)	*P*
Age, year	60.3 (10.2)	61.7 (11.5)	60.5 (11.3)	58.0 (8.4)	0.414
Male	21 (52.5%)	29 (58%)	18 (60%)	17 (40.5%)	0.294
BMI, kg/m^2^	24.2 (3.8)	23.6 (3.2)	23.5 (3.0)	24.0 (3.2)	0.781
Diabetes duration, year	NA	10 (2.5–18.3)	10.5 (5.8–20)	13 (8–20)	0.418 ^a^
Hypertension	14 (35%)	30 (60%)	15 (50%)	22 (52.4%)	0.127
CHD	1 (2.5%)	6 (12%)	5 (16.7%)	2 (4.8%)	0.118
DN	NA	4 (8%)	3 (10%)	4 (9.5%)	0.260^a^
FPG, mmol/L	4.7 (4.4–5.0)	6.8 (5.7–8.4)*	7.4 (6.2–9.5)*	5.4 (4.2–7.7) *^# ‡^	< 0.001
HbA1c, %	NA	9.4 (8.0–10.9)	8.9 (7.9–11.0)	7.3 (6.5–8.8) ^# ‡^	< 0.001^a^
HbA1c, mmol/mol	NA	79 (64–96)	74 (63–97)	56 (48–73) ^# ‡^	< 0.001^a^
Triglyceride, mmol/L	1.44 (1.00–1.93)	1.24 (0.87–1.73)	1.5 (0.99–2.03)	1.27 (0.94–1.87)	0.464
TC, mmol/L	4.45 (4.13–5.02)	4.08 (3.47–4.87)	4.22 (3.3–5.14)	4.32 (3.83–5.06)	0.295
HDL-C, mmol/L	1.10 (0.89–1.26)	0.96 (0.81–1.15)	1.02 (0.85–1.37)	1.03 (0.86–1.29)	0.226
LDL-C, mmol/L	2.59 (2.33–3.21)	2.36 (1.91–3.12)	2.12 (1.62–3.15)	2.53 (2.03–3.0)	0.277
SCr, mg/dL	0.70 (0.60–0.83)	0.62 (0.51–0.71)	0.70 (0.54–0.86)	0.65 (0.50–0.86)	0.180
eGFR, mL/min/1.73 m^2^	99.56 (91.34–104.72)	103.10 (94.42–114.64)	101.98 (85.78–112.00)	100.41 (82.96–112.48)	0.379
TMAO, µmol/L	0.357 (0.218–0.454)	0.458 (0.301–0.690)*	0.687 (0.340–1.015)*	0.793 (0.388–1.240)*^#^	< 0.001

*P* values among four or three (^a^) groups were obtained by one-way ANOVA or Kruskal–Wallis test for continuous variables and Chi-Square test for categorical variables. In post hoc pairwise comparison with Bonferroni correction, *P < 0.05 compared with control; ^#^ *P* < 0.05 compared with DWR; ^‡^ *P* < 0.05 compared with NPDR

Control, patients without diabetes; DWR, diabetes without diabetic retinopathy; NPDR, nonproliferative diabetic retinopathy; PDR, proliferative diabetic retinopathy; BMI, body mass index; CHD, coronary heart disease; DN, diabetic nephropathy; FPG, fasting plasma glucose; HbA1c, glycated hemoglobin; TC, total cholesterol; HDL-C, high density lipoprotein cholesterol; LDL-C, low density lipoprotein cholesterol; SCr, serum creatinine; eGFR, estimated glomerular filtration rate; TMAO, trimethylamine-*N*-oxide; NA, not available

### Plasma levels of TMAO in different groups

**Fig. 1:**
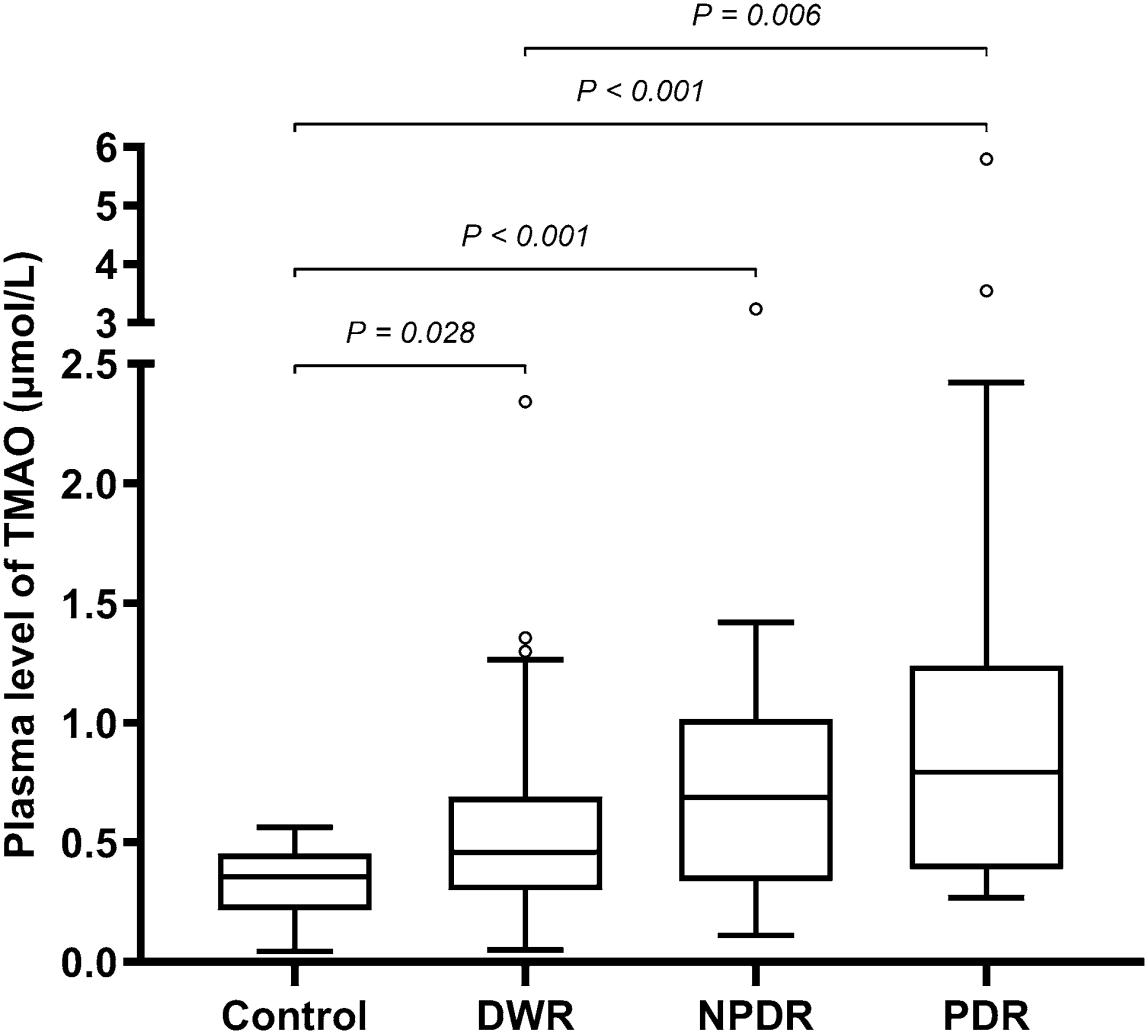
Tukey box and whisker plots display the distribution of plasma TMAO concentrations in four groups. *P* values were obtained with Kruskal–Wallis test followed by Bonferroni post hoc pairwise comparison. Box center line stands for median, and the edges of the box indicate 25th and 75th percentiles. Upper and lower whiskers show the most extreme data point that is no more than 1.5 × IQR from the edge of the box. Open circles indicate outliers in each group. TMAO, trimethylamine-*N*-oxide; Control, patients without diabetes; DWR, diabetes without diabetic retinopathy; NPDR, nonproliferative diabetic retinopathy; PDR, proliferative diabetic retinopathy; IQR, interquartile range

Tukey box and whisker plots showed the distribution of plasma TMAO concentrations (Fig. 1). The ranges (min–max) of plasma TMAO concentrations were 0.045–0.564 µmol/L, 0.051–2.342 µmol/L, 0.111–3.236 µmol/L, 0.269–5.797 µmol/L for control, DWR, NPDR, PDR groups, respectively. Kruskal–Wallis test showed a remarkable difference in plasma TMAO levels among the four groups (*P* < 0.001; Table 1). In post hoc pairwise comparison with Bonferroni correction, statistical differences in TMAO levels were observed between the control and each of the other three groups and between DWR and PDR groups. Plasma levels of TMAO in all T2DM patients were significantly higher than those of non-diabetic patients (median, 0.610 vs. 0.357 µmol/L, *P* < 0.001). All DR patients displayed higher TMAO concentrations than DWR patients (median, 0.752 vs. 0.458 µmol/L, *P* = 0.001).

The Spearman rank correlation coefficients showed that plasma levels of TMAO were positively correlated with age (*r* = 0.314, *P* < 0.001), diabetes duration (*r* = 0.233, *P* = 0.010) and SCr (*r* = 0.393, *P* < 0.001), and were negatively correlated with eGFR (*r* =  − 0.528, *P* < 0.001), but were not correlated with BMI and lipid profiles in T2DM patients (Table 2).

**Table 2:** Spearman rank correlations between plasma TMAO and variables in T2DM patients

Variables	Plasma TMAO concentrations, µmol/L	
	*R*	*P*
Age, year	0.314	< 0.001
BMI, kg/m^2^	0.131	0.151
Diabetes duration, year	0.233	0.010
Triglyceride, mmol/L	0.000	0.999
TC, mmol/L	− 0.097	0.290
HDL-C, mmol/L	− 0.046	0.614
LDL-C, mmol/L	− 0.097	0.286
SCr, mg/dL	0.393	< 0.001
eGFR, mL/min/1.73 m^2^	− 0.528	< 0.001

TMAO trimethylamine-*N*-oxide; T2DM, type 2 diabetes mellitus; BMI, body mass index; TC, total cholesterol; HDL-C, high density lipoprotein cholesterol; LDL-C, low density lipoprotein cholesterol; SCr, serum creatinine; eGFR, estimated glomerular filtration rate

### Association of plasma TMAO with DR in T2DM patients

In binary logistic regression analyses, we detected higher odds of DR as plasma TMAO quartiles increased (*P-*trend < 0.001) after adjusting the covariates (Model 2; Table 3). Patients categorized in the highest quartile of plasma TMAO were more likely to have DR compared with those in the lowest quartile (OR = 8.76; 95% CI 2.21–34.76; *P* = 0.002; Model 2; Table 3). The adjusted OR for DR was 2.62 as ln-transformed TMAO increased per SD (95% CI 1.51–4.55; *P* = 0.001; Model 2; Table 3). After further adjustment for eGFR, the results remained significant (Model 3; Table 3). Table 4 demonstrates the consistent association across the subgroup analyses. We found a statistically significant interaction between per SD increment of ln-transformed TMAO and gender (*P*-interaction = 0.006; Table 4), indicating a stronger linkage in male patients.

**Table 3:** The association of TMAO with DR in T2DM patients

	Quartiles of plasma TMAO concentrations, µmol/L				*P-*trend ^a^	Per-SD of ln (TMAO)	*P* ^b^
	Q1 (< 0.346)	Q2 (0.346–0.610)	Q3 (0.610–1.0)	Q4 (> 1.0)			
*N* (DR/DWR)	14/16	12/19	22/9	24/6		72/50	
OR (95% CI)							
Crude	1 (reference)	0.72 (0.26–1.99)	2.79 (0.97–8.03)	4.57 (1.45–14.40)	0.001	1.99 (1.29–3.07)	0.002
Model 1	1 (reference)	0.87 (0.29–2.67)	5.21 (1.52–17.87)	9.62 (2.54–36.48)	< 0.001	2.64 (1.54–4.53)	< 0.001
Model 2	1 (reference)	0.72 (0.22–2.37)	5.06 (1.39–18.46)	8.76 (2.21–34.76)	< 0.001	2.62 (1.51–4.55)	0.001
Model 3	1 (reference)	0.73 (0.22–2.41)	4.60 (1.24–17.17)	7.42 (1.72–32.00)	0.002	2.31 (1.30–4.12)	0.005

TMAO, trimethylamine-*N*-oxide; T2DM, type 2 diabetes mellitus; DR, diabetic retinopathy; DWR, diabetes without diabetic retinopathy; BMI, body mass index; Q, quartile; SD, standard deviation; ln, logarithmic; OR, odds ratio; CI, confidence interval

^a^*P* values for linear trend across quartiles of TMAO were calculated by treating the median of each quartile as a continuous variable in binary logistic regression

^b^*P* values were obtained by binary logistic regression analyses. Model 1 was adjusted for age and gender. Model 2 was adjusted for age, gender, BMI, diabetes duration and hypertension. Model 3 was adjusted for covariates included in Model 2 plus estimated glomerular filtration rate

**Table 4:** Adjusted ORs for TMAO related to DR in T2DM patients across subgroups

Subgroups	OR (95% CI) ^a^	*P*	*P*-interaction
Age, year			0.098
≤ 60	2.76 (1.28–5.97)	0.010	
> 60	2.65 (1.07–6.57)	0.035	
Gender			0.006
Male	4.17 (1.73–10.04)	0.001	
Female	2.13 (0.97–4.65)	0.059	
BMI, kg/m^2^			0.108
≤ 24	3.35 (1.42–7.91)	0.006	
> 24	2.31 (1.04–5.09)	0.039	
Diabetes duration, year			0.159
≤ 11	3.57 (1.55–8.23)	0.003	
> 11	2.11 (0.96–4.61)	0.063	
Hypertension			0.101
No	3.53 (1.32–9.43)	0.012	
Yes	2.25 (1.09–4.65)	0.029	

TMAO, trimethylamine-*N*-oxide; T2DM, type 2 diabetes mellitus; DR, diabetic retinopathy; BMI, body mass index; OR, odds ratio; CI, confidence interval; SD, standard deviation; ln, logarithmic

^a^ORs and their corresponding 95% CI per SD increment in the ln-transformed TMAO were obtained by binary logistic regression adjusted for age, gender, BMI, diabetes duration and hypertension

In univariate ordinal logistic regression model, we found that per SD increment of ln-transformed TMAO was correlated with worse DR severity (OR = 1.92; 95% CI 1.32–2.80; *P* = 0.001). After adjusting the covariates included in Model 3, this association remained significant (OR = 2.05; 95% CI 1.27–3.33; *P* = 0.004).

### Plasma TMAO in DR diagnosis in T2DM patients

**Fig. 2:**
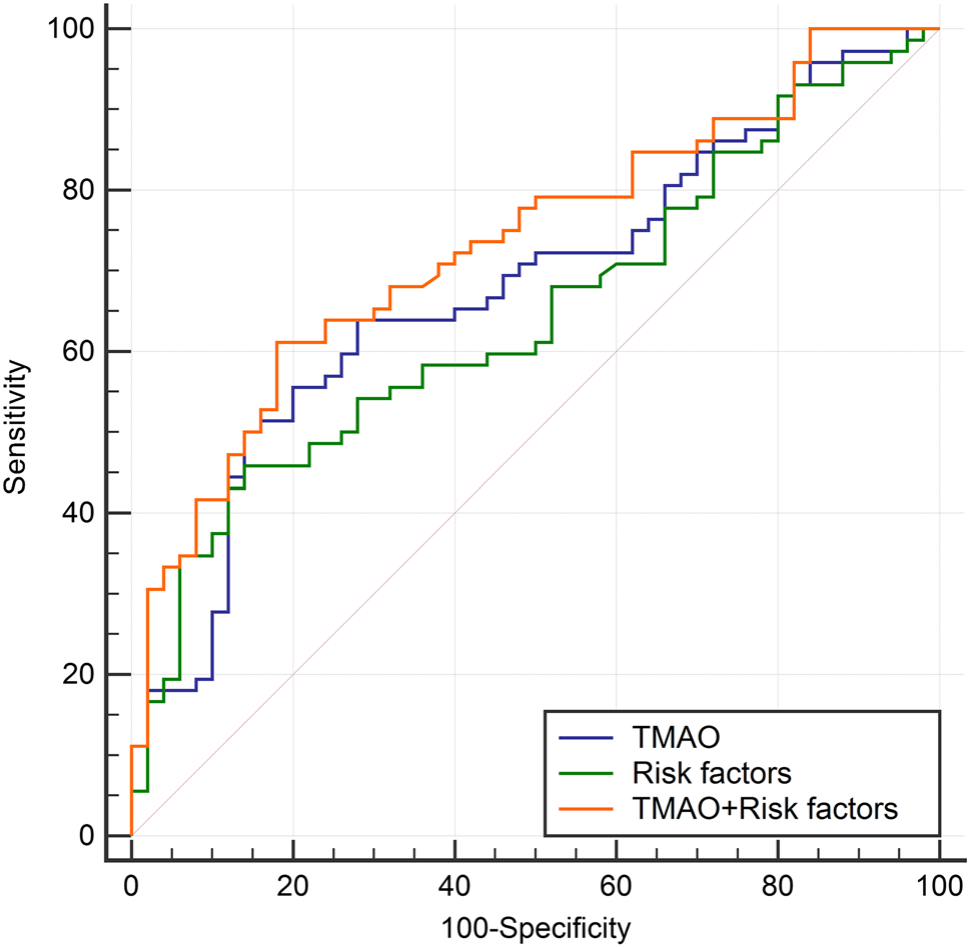
ROC for three models to DR diagnosis in T2DM patients. Blue line indicates TMAO alone; green line indicates risk factors model including age, gender, BMI, diabetes duration and hypertension; orange line indicates risk factors model plus TMAO. ROC, receiver operating curves; DR, diabetic retinopathy; T2DM, type 2 diabetes mellitus; TMAO, trimethylamine-*N*-oxide; BMI, body mass index

As shown in Fig. 2, the AUROC for TMAO alone was 0.679 (95% CI 0.589–0.761; *P* < 0.001) with optimal cutoff value of 0.761 µmol/L yielded 50% sensitivity and 86% specificity. The AUROC was 0.646 (95% CI 0.555–0.731; *P* = 0.003) for the primary diagnostic model and 0.734 (95% CI 0.647–0.810; *P* < 0.001) for a new model combining the acknowledged factors and TMAO, indicating statistical improvement in AUROC due to the addition of TMAO as 0.088 (95% CI 0.003–0.173; *P* = 0.043). We also found that the addition of TMAO resulted in improvement of discriminatory power of the model (IDI = 10.7%; 95% CI 5.3–16.1%; *P* < 0.001).

## Discussion

In the present study, we found a novel relationship between plasma TMAO and DR, which has not been reported before as far as we know. Plasma levels of TMAO significantly elevated in DR patients compared with DWR patients and were correlated with DR severity. Our results provided preliminary evidence supporting that higher plasma TMAO levels were independently associated with increased odds of DR in T2DM patients. Prospective cohort studies with larger sample are required to further determine the causal relationship.

It was surprising that age and diabetes duration showed no significant difference among DWR, NPDR and PDR groups, and FPG and HbA1c levels in PDR group were lower than those in DWR and NPDR groups in this cross-sectional, hospital-based study. These results were inconsistent with previous reports [19, 20] and might mainly arise from limited sample size and selection bias. Patients of DWR and NPDR were in hospital due to poor glycemic control, and most of them were not newly diagnosed early-onset diabetic patients. In addition, time points for blood sampling might also be a reason for lower FPG and HbA1c levels in PDR group, because blood glucose had been well controlled in almost all PDR patients before admission for vitrectomy. Besides, previous study found that there was no significant association of TMAO with changes in FPG and HbA1c [10]. In view of this, we did not include FPG and HbA1c into subsequent analyses.

Our quantitative analyses revealed lower plasma TMAO concentrations in the subjects of Chinese Han ethnicity compared with Western population [4]. This might due to the distinct dietary. Omnivores with higher fat diet reportedly produced more TMAO than vegetarians due to their different gut microbiota [21]. Despite our exclusion of vegetarians, the Chinese diet containing less animal products and more vegetables was still more likely to result in lower plasma TMAO.

Recent studies suggested that increased plasma TMAO portended more CVD events, worse renal outcome, and higher mortality risk independent of glycemic control and other conventional risk factors for diabetes [11, 22, 23]. Our study showed higher plasma levels of TMAO in T2DM patients than non-diabetic patients, and this result was in line with previous study [17], indicating the important role of TMAO in diabetes and its complications. Spearman rank correlation analyses showed positive correlation between TMAO and age, as well as diabetes duration. We also observed a correlation between TMAO and renal function parameters—positive with SCr and negative with eGFR in T2DM patients. These results are rational since some studies based on humans and mice have reported similar results before [11, 23–25].

Logistic regression analysis showed the relevance between TMAO and presence of DR in T2DM patients. All of the univariate and multivariate results demonstrated that patients categorized into the highest quartile of plasma TMAO concentrations were more prone to DR. Odds of DR approximately doubled as the ln-transformed TMAO increased per SD. Beyond that, this association was found stronger in male patients through subgroups analyses. The interaction effect between plasma TMAO and gender may be partly explained by the sex differences in dietary pattern, gut microbiome composition [26, 27], but it still requires further validation of the interaction and investigation of potential mechanisms.

When analyzing the plausibility of applicating plasma levels of TMAO into DR diagnosis, the results showed low value of TMAO alone for DR diagnosis. However, through comparison of AUROC and calculation of IDI, we found that the discriminative ability of the model improved after addition of TMAO into the model with known risk factors. These results indicated an application prospect of plasma TMAO in clinical management of DR, and future study should make further efforts to confirm the application value of TMAO by enrollment of a larger population.

Our study detected a relationship between plasma TMAO and presence of DR, but up to date, the exact causal direction of this association remains unclear. It was indicated that diabetic gut microbiota dysbiosis could promote DR development [28], and gut microbial compositions in DWR or DR patients were different from that in controls [29]. Beli et al. found that intermittent fasting could prevent the progression of DR by restructuring the gut microbiome with increased Firmicutes and decreased Bacteroidetes [30]. Levels of plasma TMAO were also reported negatively correlated with Firmicutes and positively linked with Bacteroidetes at the phylum levels in mice [31]. We tried our best to speculate several possible mechanisms basing on previous studies discussing the pathophysiological roles of TMAO in other diabetes-related or cardiovascular diseases.

Firstly, TMAO might take part in DR development by enhancing insulin resistance and dyslipidemia. As noted previously, higher levels of plasma TMAO were correlated with greater insulin resistance and less improvement of the diabetes-related outcomes [10, 32]. Besides, FMO3 was suggested as a central regulator of cholesterol balance involving liver X receptor (LXR) activity [33], which played a key role in protection against DR [34].

Secondly, systemic inflammation might be another mechanism. Inflammation serves as a pivotal mechanism governing the progression of DR [35]. Elevated plasma levels of TMAO could lead to a mild and chronic systemic inflammatory state with higher plasma pro-inflammatory cytokines [36]. Mice fed in Western diet had greater plasma TMAO concentrations, with increased levels of TNF-*α* and IL-1*β* and decreased levels of IL-10 [37].

Thirdly, vascular inflammation and endothelial dysfunction could be involved. TMAO could directly activate inflammatory signaling pathway and facilitate recruitment of leukocytes [38, 39]. It was also suggested that TMAO could accelerate endothelial cell senescence through promoting excessive reactive oxygen species (ROS) production and inhibiting Sirtuin1 [24]. Recent study has also uncovered that high mobility group box 1 (HMGB1) was involved in TMAO-induced vascular endothelial hyper-permeability [40].

However, several limitations should be considered when interpreting our results. Firstly, the cross-sectional design might enhance selection and recall bias, and our study could not identify the causal direction between TMAO and DR. Secondly, our limited sample size caused insufficient representativeness of the general population as well as large confidence intervals in logistic regression analyses. Thirdly, we did not use nutritional epidemiology methods to record detail dietary pattern and food intake data, and we did not examine plasma choline, TMA and composition of intestinal flora, which were related to TMAO generation. Fourthly, aqueous and vitreous levels of TMAO were not evaluated in our study, and future study should compare the TMAO concentrations across different body fluid. Finally, different lifestyle factors and comorbidities relevant to the disease were not considered, which might potentially influence the results.

In conclusion, our study demonstrated elevated plasma levels of TMAO associated with higher odds and worse severity of DR in T2DM patients. Further studies with prospective cohort study design are warranted to establish causal relationship and to elucidate the exact molecular mechanisms underlying this association in detail.

## Data Availability

The datasets generated during and/or analyzed during the current study are available from the corresponding author on reasonable request.
